# The Effect of *APOE* ε4 Allele on Dynamic Local Spontaneous Brain Activity and Functional Integration in Alzheimer's Disease

**DOI:** 10.1002/hbm.70269

**Published:** 2025-07-11

**Authors:** Yi Tan, Dan Yang, Zhihong Ke, Zheqi Hu, Wenting Song, Limoran Tang, Zhixin Zhou, Yuting Mo, Lili Huang, Yun Xu

**Affiliations:** ^1^ Department of Neurology Nanjing Drum Tower Hospital, Affiliated Hospital of Medical School, Nanjing University Nanjing China; ^2^ Department of Neurology Nanjing Drum Tower Hospital, Clinical College of Nanjing Medical University Nanjing China; ^3^ Department of Neurology Nanjing Drum Tower Hospital, State Key Laboratory of Pharmaceutical Biotechnology and Institute of Translational Medicine for Brain Critical Diseases, Nanjing University Nanjing China; ^4^ Jiangsu Key Laboratory for Molecular Medicine, Medical School of Nanjing University Nanjing China; ^5^ Jiangsu Provincial Key Discipline of Neurology Nanjing China

**Keywords:** Alzheimer's disease, apolipoprotein E ε4, dynamic local spontaneous activity, resting‐state functional magnetic resonance imaging, voxel‐wise concordance

## Abstract

The apolipoprotein E (*APOE*) ε4 allele is the most important genetic risk factor for sporadic Alzheimer's disease (AD), yet its mechanisms in AD pathology and cognitive decline remain unclear. Using a sliding‐time window approach to directly quantify the instantaneous fluctuations of various local metrics based on continuous time series and calculate voxel‐wise concordance of these metrics, we explored the impact of *APOE* ε4 on dynamic local brain activity and functional integration in AD, and its interrelations with plasma biomarkers and cognition. Results showed that *APOE* ε4 widely affected dALFF, dReHo, dGSCorr, and voxel‐wise concordance. For AD patients, *APOE* ε4 carriers uniquely exhibited correlations between dALFF in the right angular gyrus/supramarginal gyrus and MoCA scores and orientation function, and between voxel‐wise concordance in the right caudate nucleus (CAU) and general cognition, attention, language function, orientation function, plasma Aβ42. Critically, *APOE* ε4‐related altered voxel‐wise concordance in the right CAU mediated the relationship between plasma Aβ and language cognition in AD. Moreover, the combined model incorporating dynamic metrics, plasma AD biomarkers, and demographic data effectively distinguished AD from NC (AUC = 0.94, sensitivity = 87.69%, specificity = 86.84%). In conclusion, the *APOE* ε4 allele might play a pivotal role in modulating brain dynamic functional activities in AD, which may contribute to the association between Aβ pathology and cognitive decline. Our findings may provide imaging markers and targets for the diagnosis and treatment of AD.

## Graphical abstract

The voxel‐wise concordance in the right CAU partially mediated the relationship between the plasma Aβ42 and language function in *APOE* ε4 carriers with AD.

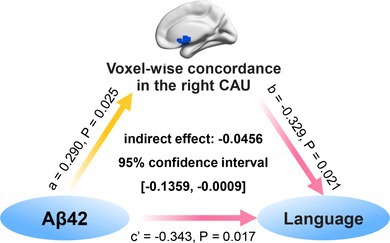

**Abbreviations**
AD: Alzheimer's disease
ANG: angular gyrus
*APOE*
: apolipoprotein E
AUC: area under the curve
Aβ: amyloid beta
CAL: calcarine fissure and surrounding cortex
CAU: caudate nucleus
CI: confidence interval
CUN: cuneus
CV: coefficient of variation
dALFF: dynamic amplitude of low frequency fluctuations
dfALFF: dynamic fractional ALFF
dGSCorr: dynamic global signal correlation
dReHo: dynamic regional homogeneity
FC: functional connectivity
GFAP: glial fibrillary acidic protein
IF Goperc: opercular part of inferior frontal gyrus
IF Gtriang: triangular part of inferior frontal gyrus
IPL: inferior parietal lobe
MCI: mild cognitive impairment
MMSE: Mini‐Mental State Examination
MoCA: Montreal Cognitive Assessment
NC: normal cognition
NfL: neurofilament light chain
OL: occipital lobe
PCUN: precuneus
p‐tau: phosphorylated tau
ROC: receiver operating characteristic
rs‐fMRI: resting‐state functional magnetic resonance imaging
SD: standard deviation
SMG: supramarginal gyrus
SPG: superior parietal gyrus
TIV: total intracranial volume
TR: repetition time

**Summary**

- The *APOE* ε4 allele widely impacted dynamic local spontaneous brain metrics including dALFF, dReHo, and dGSCorr, and voxel‐wise concordance of these metrics in AD.
- The *APOE* ε4 allele influenced the correlations of dynamic functional activities with cognition and plasma Aβ pathology in AD.
- The voxel‐wise concordance in the right caudate nucleus partially mediated the relationship between plasma Aβ42 and language function in *APOE* ε4 carriers with AD.
- The combined model incorporating dynamic metrics, plasma AD biomarkers, and demographic data effectively distinguished AD from NC.

## 1 Introduction

Alzheimer's disease (AD), a progressive neurodegenerative disease with insidious onset, is the main cause of dementia (Scheltens et al. 2021). AD involves dysregulation of complex cellular and molecular processes (Knopman et al. 2021) and is associated with multiple genetic variations (Schwartzentruber et al. 2021). Among these, the apolipoprotein E (*APOE*) ε4 allele is the most important genetic risk factor for sporadic ad (Serrano‐Pozo et al. 2021; Uebergang et al. 2023) and plays an important role in AD pathological processes (Haney et al. 2024; Montagne et al. 2020; Blanchard et al. 2022). By age 85, the lifetime risk for AD in *APOE* ε4 homozygotes reaches 60%, and heterozygotes exhibit semi‐dominant effects of this moderately penetrant gene (Fortea 2024; Genin et al. 2011). However, the mechanism by which the *APOE* ε4 allele mediates AD pathology remains incompletely understood. Therefore, exploring the underlying pathogenesis of the *APOE* ε4 allele may provide new therapeutic opportunities for AD patients.

Over the past decades, resting‐state functional magnetic resonance imaging (rs‐fMRI) has been widely used to explore functional abnormalities in the brains of *APOE* ε4 carriers. The most frequently applied functional metrics include functional connectivity (FC) and local spontaneous brain activity metrics. For example, in healthy human adults, *APOE* ε4 carriers exhibited increased lateralized connections associated with callosal connections within the default mode, memory, and salience networks compared to non‐carriers (Butt et al. 2022). Decreased FC in the left hippocampus of *APOE* ε4 carriers was associated with verbal memory decline (Baxter et al. 2023). Regarding local brain activity, *APOE* ε4 carriers with AD exhibit decreased amplitude of low frequency fluctuations (ALFF) in the left hippocampus (Wang et al. 2015) and decreased fractional ALFF (fALFF) in the left insula, left inferior frontal gyrus, and right precentral gyrus (Lin et al. 2017) compared to non‐carriers. Additionally, cognitively normal young adult *APOE* ε4 carriers showed increased regional homogeneity (ReHo) in the right superior frontal gyrus which correlated with neuropsychological test scores (Zheng et al. 2017); Cai 2017). also reported altered ALFF and ReHo in the anterior cingulate cortex, medial prefrontal cortex, and precuneus in *APOE* ε4 carriers with normal cognition (NC), indicating gray matter activity changes in these regions (Cai 2017). Notably, the plasma AD biomarkers such as amyloid beta (Aβ), glial fibrillary acidic protein (GFAP) (Lohman et al. 2024), neurofilament light chain (NfL) (Lohman et al. 2024), and phosphorylated tau (p‐tau) (Zhu et al. 2024) have been associated with *APOE* ε4‐related functional changes.

However, given that the human brain remains active during the resting state, static rs‐fMRI metrics described previously could not reflect temporal characteristics of local brain activity in *APOE* ε4 carriers. Fortunately, compared to other dynamic fMRI approaches, such as point process analysis and time frequency analysis, the sliding‐time window method directly quantifies the instantaneous fluctuations of various local rs‐fMRI metrics from continuous time series (Yang et al. 2024; Chen et al. 2023). Since dynamic metrics reflect brain activity from different perspectives, the voxel‐wise concordance derived from them may reveal the interaction among these metrics across states. The sliding‐window approach enables calculation of voxel‐level synchronization of multiple dynamic local metrics, objectively and comprehensively reflecting the mechanism of brain function integration (Yan 2017). A previous study has shown lower global voxel‐wise concordance in patients with AD than in healthy controls (Chen et al. 2023). However, no studies have investigated the effect of the *APOE* ε4 allele on AD from the perspective of local dynamic metrics and their integration. Therefore, investigating the impact of the *APOE* ε4 allele on these metrics in AD populations may provide novel insights into AD pathophysiological mechanisms unobtainable from static or single‐metric analyses.

In this study, we compared dynamic ALFF (dALFF), dynamic fALFF (dfALFF), dynamic ReHo (dReHo), and dynamic global signal correlation (dGSCorr), and voxel‐wise concordance of these dynamic metrics across four groups stratified by *APOE* ε4 allele and cognitive status. We subsequently investigated (1) the effect of *APOE* ε4 allele on correlations between dynamic metrics and cognition, plasma AD biomarkers in AD; (2) the mediation effect of *APOE* ε4‐related alterations in dynamic metrics on the relationship between Aβ pathology and cognitive function; (3) the diagnostic performance of combined models in identifying AD patients.

## 2 Materials and Methods

### 2.1 Participants

The current study was conducted under the latest version of the Declaration of Helsinki and authorized by the Review board of Nanjing Drum Tower Hospital (Ethical Approval Code: 2022–472‐01). Informed consent was obtained from all participants. All participants were recruited from two medical centers and local communities. Among them, 7 participants were excluded due to excessive head motion (cumulative translation or rotation of > 3.0 mm or 3.0°). 418 individuals were ultimately included in this study. All participants completed *APOE* genotyping tests and 3.0‐T whole brain MRI scanning, 417 participants completed the Mini‐Mental State Examination (MMSE), 396 participants completed the Montreal Cognitive Assessment (MoCA), 377 participants completed measurements of plasma AD biomarkers including Aβ42, Aβ40, GFAP, NfL, and p‐tau181. Age, sex, and years of education were collected as demographics. Hypertension, diabetes mellitus, and hyperlipidaemia were considered as risk factors. Each participant's blood collection, cognitive tests, and image acquisition were conducted within 1 month.

Dementia and mild cognitive impairment (MCI) were identified by education‐adjusted norms of MMSE and MoCA (Katzman et al. 1988; Gorelick et al. 2011; Lu et al. 2011), respectively. The cut‐off scores of MMSE and MoCA for different education levels were clearly described in previous study (Yang et al. 2023). Dementia and MCI due to probable AD were diagnosed in accordance with the recommendations of the National Institute on Aging‐Alzheimer's Association (Albert et al. 2011; McKhann et al. 2011). Subjects with dementia and MCI due to probable AD were classified into the AD group. *APOE* ε4 carriers were defined as individuals with at least one ε4 allele. All participants were divided into 160 *APOE* ε4 non‐carriers with NC, 38 *APOE* ε4 carriers with NC, 155 *APOE* ε4 non‐carriers with AD, and 65 *APOE* ε4 carriers with AD.

The exclusion criteria for both NC and AD groups were as follows: (1) ≤ 50 years old; (2) central nervous system diseases that could cause cognitive impairment, such as vascular cognitive impairment, Parkinson's disease dementia, and dementia with Lewy body; (3) a history of stroke and subarachnoid hemorrhage; (4) other severe neurological diseases, such as brain tumor, epilepsy, and multiple sclerosis; (5) psychiatric diseases, such as major depressive disorder, bipolar affective disorder, autistic, and schizophrenia; (6) severe systemic diseases, such as cancer and heart failure; (7) alcoholism.

### 2.2 Cognitive Assessment

General cognition was evaluated by both MMSE and MoCA scores. Scores of cognitive domains of MoCA were calculated. Among them, executive function was evaluated using the sum score of Alternating Trail Making, Visuoconstructional Skills (cube), and Visuoconstructional Skills (clock). Attention was evaluated using the sum score of Forward Digit Span, Backward Digit Span, Vigilance, and Serial 7 s. Language function was evaluated using the sum score of Sentence repetition and Verbal fluency. All cognitive examinations were completed by two professional neuropsychologists.

### 2.3 MRI Data Acquisition

All MRI scans were performed on a Philips Medical Systems 3.0 T scanner. The rs‐fMRI data were collected with the following parameters: high‐resolution 3D T1 imaging: echo time (TE) = 4.6 ms, repetition time (TR) = 9.8 ms, flip angle (FA) = 8°, field of view (FOV) = 250 × 250 mm^2^, number of slices = 192, matrix size = 192 × 256 × 256, thickness = 1.0 mm; rs‐fMRI was acquired by a gradient‐echo‐planar imaging sequence: TE = 30 ms, TR = 2000 ms, FA = 90°, FOV = 192 × 192 mm^2^, matrix size = 64 × 64, thickness = 4.0 mm, number of slices = 35, and each functional image contained 240 volumes. Besides, T2‐weighted and diffusion‐weighted imaging were obtained to exclude acute or sub‐acute infarctions. All participants were asked to keep their eyes closed, not to move, not to sleep, and not to think about anything during scanning.

### 2.4 Volume Quantification

The total intracranial volume (TIV) was calculated with the Statistical Parametric Mapping 8 (SPM8, http://www.fil.ion.ucl.ac.uk/spm) based on 3D‐T1 images.

### 2.5 Rs‐fMRI Data Preprocessing

Original functional images were preprocessed with a standard pipeline in the toolbox for data processing and analysis of brain imaging (DPARSF, V3.2, http://www.restfmri.net) and SPM 12 (http://www.fil.ion.ucl.ac.uk/spm). The specific process was as follows: (1) The first 10 volumes of data were removed; (2) The slice timing correction and realignment were conducted on the remaining volumes; (3) The re‐alignment was performed to correct the movement between time points; (4) Head motion parameters were calculated by evaluating the translation in each direction and the angular rotation on each axis for each volume, then individuals with cumulative translation > 3.0 mm or rotation > 3.0° were excluded; (5) Single T1‐weighted images were segmented into gray matter, white matter, and cerebrospinal fluid and co‐registered to the mean functional image through a 6° freedom linear transformation without re‐sampling; (6) Linear trends, Friston 24 head motion parameters, the white matter signal and cerebrospinal fluid signal were regressed out from the functional signal as nuisance covariates to reduce head motion artifacts. The global signal regression was not performed due to the attendant controversy (Murphy and Fox 2017); (7) The Diffeomorphic Anatomical Registration Through Exponentiated Lie algebra (DARTEL) tool was used to generate transformations from individual native space to the standard Montreal Neurological Institute (MNI) space (3 × 3 × 3 mm^3^); (8) For ReHo and GSCorr, bandpass filters (0.01 Hz < f < 0.1 Hz) were applied.

### 2.6 Calculation of Dynamic Local Metrics

(1) ALFF (Yu‐Feng et al. 2007)/fALFF (Zou et al. 2008): ALFF and fALFF were used to measure amplitude by transforming the blood oxygen level dependent time course to the frequency domain via fast Fourier transform. ALFF was computed as the mean of amplitudes within a specific low‐frequency range (0.01–0.1 Hz). fALFF was the ratio of the sum of amplitudes of a given low frequency band (0.01–0.1 Hz) to that across the entire frequency range. In this study, fALFF was chosen to calculate voxel‐wise concordance because of the high co‐linearity between these two metrics and the sensitivity and specificity of fALFF in spontaneous brain activity (Yan et al. 2013; Zuo et al. 2010).

(2) ReHo (Zang et al. 2004): ReHo was used to assess the degree of regional coherence. It was the Kendall's coefficient of concordance or Kendall's W of the time series of a given voxel with those of its nearest neighbors (27 voxels).

(3) GSCorr (Hahamy et al. 2014): GSCorr was calculated as the Pearson's correlation coefficient between the global average signal and each voxel signal within the gray matter mask. These correlation values were then Fisher‐Z transformed.

Dynamic local metrics were calculated via sliding time window method in the Data Processing and Analysis of Brain Imaging (DPABI, V6.1, http://rfmri.org/DPABI). Specifically, the sliding window size of 30 TR and sliding step of 1 TR was first applied to perform window time series analyses, resulting in 181 windows for each subject. Within each window, dynamic local metrics were calculated in a voxel‐wise way. The standard deviation (SD), coefficient of variation (CV), and mean maps of these dynamic metrics were then generated. The SD maps were used to assess stability of each metric, CV maps were used to assess variability of each metric, and mean maps were used to quantify the temporal dynamic characteristics. There was no CV map of dGSCorr because it contained negative values. Finally, these SD, CV, and mean maps were subjected to z‐standardization and smoothed with a 4 × 4 × 4 mm^3^ full‐width at half maximum (FWHM) Gaussian Kernel.

### 2.7 Calculation of Voxel‐Wise Concordance

Voxel‐wise concordance was obtained from the Kendall's W of dfALFF, dReHo, and dGSCorr across time windows for each voxel. Maps of voxel‐wise concordance were transformed with z‐standardization to reduce the effect of variability of global activities among subjects. These maps were then spatially smoothed with a Gaussian kernel of 4 × 4 × 4 mm^3^ FWHM.

To verify the robustness of this study, results using different window lengths (window length = 40 TR, window step = 1 TR) as well as different window steps (window length = 30 TR, window step = 2 TR) were analyzed.

### 2.8 Statistical Analysis

Normality of continuous variables was assessed by the Shapiro–Wilk test. The Levene's test was used to examine the homogeneity of variance. One‐way analyses of variance (ANOVA) tests and Kruskal‐Wallis H‐tests were used to compare continuous numerical variables across the four groups, as appropriate. A Chi‐squared test was used to compare the distribution of sex among the four groups. Bonferroni correction and Kruskal‐Wallis ANOVA were used as post hoc tests for characteristics at baseline. *p* < 0.05 was considered statistical significance. All the above statistical analyses were conducted in the SPSS software (version 22.0; IBM Corporation, Armonk, NY, USA). Based on the results of the baseline analysis, age, sex, and years of education were included as covariates in all the following analyses to reduce the impact of confounding variables.

Mixed effect analyses of dynamic rs‐fMRI metrics were performed by DPABI (V6.1, http://rfmri.org/DPABI) within a gray matter mask (GreyMask_02_61 × 73 × 61), with age, sex, and years of education as covariates. *APOE* ε4 status was considered a group effect variable, while cognitive status was considered a condition effect variable. Multiple comparisons correction was performed using Gaussian Random Field (GRF) theory. The voxel‐level threshold was *p* < 0.001 for dALFF, dfALFF, dReHo, and dGSCorr, and was *p* < 0.01 for voxel‐wise concordance. The cluster‐level threshold was *p* < 0.05. For each metric, a cluster size threshold of 40 voxels was applied. Finally, the values of each cluster were extracted. ANOVA tests followed by Bonferroni correction were then used to compare values of these metrics across the four groups.

To explore the effect of *APOE* ε4 allele on relationship between dynamic rs‐fMRI metrics and cognition, plasma AD pathology, partial correlation analyses were performed in both *APOE* ε4 carriers and non‐carriers in AD group, adjusting for age, sex, and years of education.

To further explain the interrelations amongst Aβ42, voxel‐wise concordance, and cognition, mediation analyses were conducted among *APOE* ε4 carriers with AD, adjusting for age, sex, and years of education. The bootstrapping (*k* = 5000 samples) in PROCESS for SPSS 22.0 was used to obtain the bias‐corrected 95% confidence interval (CI) for the mediating effect. The mediating effect was considered statistically significant if the 95% CI did not contain the value 0.

Finally, receiver operating characteristic curve (ROC) analysis was used to evaluate the discriminative ability of metric models in identifying AD patients under distinct *APOE* ε4 status. Model 1 was the combination of age, sex, years of education, and *APOE* ε4‐related alterations in dynamic metrics. Model 2 was the combination of age, sex, years of education, and *APOE* ε4‐related and cognition‐related alterations in dynamic metrics. Model 3 was the combination of age, sex, years of education, AD plasma biomarkers, and *APOE* ε4‐related and cognition‐related alterations in dynamic metrics. To prevent model overfitting, we conducted cross‐validation analysis with k‐fold cross‐validation (*k* = 5) (Poldrack et al. 2020; White and Power 2023). Specifically, the data were randomly divided into 5 subgroups. The holdout approach was then repeated 5 times, with one of the 5 subsets providing the test set and the other 4 subsets forming a training set each time. The performance estimation was averaged over all 5 trials to evaluate overall efficiency. All the above analyses were conducted in the R software (version 4.4.1).

## 3 Results

### 3.1 Demographic, Risk Factors, Cognitive, Pathologic, and Imaging Characteristics

Overall, as shown in Table 1, there were 38 (19.19%) and 65 (29.55%) *APOE* ε4 carriers in NC and AD groups, respectively. There were no group differences in sex distribution, plasma Aβ40, plasma NfL, TIV, or prevalence of hypertension, diabetes mellitus, and hyperlipidaemia across the four groups (all *p* > 0.05). Compared with the NC group, subjects with AD showed older age and lower education level (Bonferroni and Kruskal‐Wallis ANOVA corrected, *p* < 0.05). Furthermore, within the AD group, as compared to non‐carriers, *APOE* ε4 carriers showed worse orientation function, lower plasma Aβ42 and plasma Aβ42/40 ratio, higher plasma GFAP, and plasma p‐tau181 (Bonferroni and Kruskal‐Wallis ANOVA corrected, *p* < 0.05).

**TABLE 1:** Summary of demographic, risk factors, cognitive, pathologic and imaging characteristics across the four groups.

	Ɛ4 non‐carriers with NC	Ɛ4 carriers with NC	Ɛ4 non‐carriers with AD	Ɛ4 carriers with AD	*p*
	*n* = 160	*n* = 38	*n* = 155	*n* = 65	
Demographics					
Age (years)	66.68 ± 8.16	65.92 ± 10.27	69.79 ± 8.70^d^, ^e^	70.66 ± 8.97^d^, ^e^	0.001^a^
Female (*n*, %)	80 (50.0)	25 (65.8)	91 (58.7)	35 (53.8)	0.228^b^
Education level (years)	12 (9,15)	12 (8.25,16)	9 (7,12)^d^, ^e^	12 (6,15)	< 0.001^c^
Risk factors					
Hypertension (*n*, %)	78 (48.8)	17 (44.7)	69 (44.2)	35 (53.8)	0.611^b^
Diabetes mellitus (*n*, %)	47 (29.4)	12 (32.6)	47 (30.3)	20 (30.8)	0.828^b^
Hyperlipidaemia (*n*, %)	67 (41.9)	15 (39.5)	62 (40.0)	25 (38.5)	0.627^b^
General cognition					
MMSE	29 (28,30)	29 (28,30)	25 (22,28)^d^, ^e^	22 (15,26)^d^, ^e^	< 0.001^c^
MoCA	26 (25,28)	26 (24.75,28)	19 (15,22)^d^, ^e^	19 (12,21)^d^, ^e^	< 0.001^c^
Cognitive domains					
Executive function	5 (4,5)	4 (3,4.75)	3 (2,3)^d^, ^e^	2 (1,4)^d^, ^e^	< 0.001^c^
Naming ability	2 (2,3)	2 (2,3)	1 (1,2)^d^, ^e^	1 (1,2)^d^, ^e^	< 0.001^c^
Attention	6 (6,6)	6 (6,6)	6 (5,6)^d^, ^e^	5.5 (4,6)^d^, ^e^	< 0.001^c^
Language function	3 (2,3)	3 (2.25,3)	2 (1,3)^d^, ^e^	2 (1,3)^d^, ^e^	< 0.001^c^
Abstraction	2 (1,2)	2 (1,2)	1 (0,1)^d^, ^e^	1 (1,2)^d^, ^e^	< 0.001^c^
Delayed recall	3 (2,4)	3 (2,4)	0 (0,2)^d^, ^e^	0 (0,1.25)^d^, ^e^	< 0.001^c^
Orientation function	6 (6,6)	6 (6,6)	6 (4.75,6)^d^, ^e^	5 (3,6)^d^, ^e^, ^f^	< 0.001^c^
AD plasma biomarkers					
Aβ42 (pg/ml)	6.94 ± 2.06	6.81 ± 1.77	6.76 ± 1.85	6.09 ± 1.86^d^, ^e^	0.041^a^
Aβ40 (pg/ml)	103.30 ± 22.73	102.65 ± 18.95	101.31 ± 20.92	102.49 ± 24.49	0.901^a^
Aβ42/40 ratio	0.068 ± 0.016	0.067 ± 0.016	0.067 ± 0.013	0.060 ± 0.012^d^, ^e^, ^f^	0.003^a^
GFAP (pg/ml)	118.82 ± 54.84	122.86 ± 55.19	156.21 ± 150.45	210.26 ± 151.06^d^, ^e^, ^f^	< 0.001^c^
NfL (pg/ml)	24.29 ± 32.92	20.79 ± 18.23	27.91 ± 29.22	28.88 ± 17.84	0.398^a^
p‐tau181 (pg/ml)	2.04 ± 1.04	2.18 ± 0.84	3.18 ± 1.97^d^	4.23 ± 2.52^d^, ^e^, ^f^	< 0.001^c^
Imaging data					
TIV (ml)	1354.72 ± 116.93	1333.91 ± 128.49	1333.48 ± 125.55	1324.53 ± 111.51	0.262^a^

*Note:* Continuous numerical variables were given as mean ± SD, discontinuous numerical variables were given as median (interquartile range), and categorical variables were given as integer (percentage). MMSE was available in 417 (99.76%) participants. MoCA was available in 396 (94.74%) participants. Executive function was available in 328 (77.51%) participants. Naming ability was available in 329 (77.51%) participants. Attention was available in 330 (77.27%) participants. Language function was available in 331 (79.18%) participants. Abstraction was available in 332 (77.51%) participants. Delayed recall was available in 323 (77.27%) participants. Orientation function was available in 324 (77.51%) participants. Plasma AD biomarkers were available in 377 (90.19%) participants.

Abbreviations: Aβ, amyloid beta; GFAP, glial fibrillary acidic protein; MMSE, Mini‐Mental State Examination; MoCA, Montreal Cognitive Assessment test; NfL, neurofilament light chain; p‐tau, phosphorylate tau; TIV, total intracranial volume.

^a^ One‐way ANOVA test.

^b^ Chi‐squared test.

^c^ Kruskal‐Wallis H‐test.

^d^ Post hoc analysis showed a significant group difference compared to Ɛ4 non‐carriers with NC.

^e^ Post hoc analysis showed a significant group difference compared to Ɛ4 carriers with NC.

^f^ Post hoc analysis showed a significant group difference compared to Ɛ4 non‐carriers with AD.

### 3.2 Alterations of Dynamic Local Metrics and Voxel‐Wise Concordance

**FIGURE 1:**
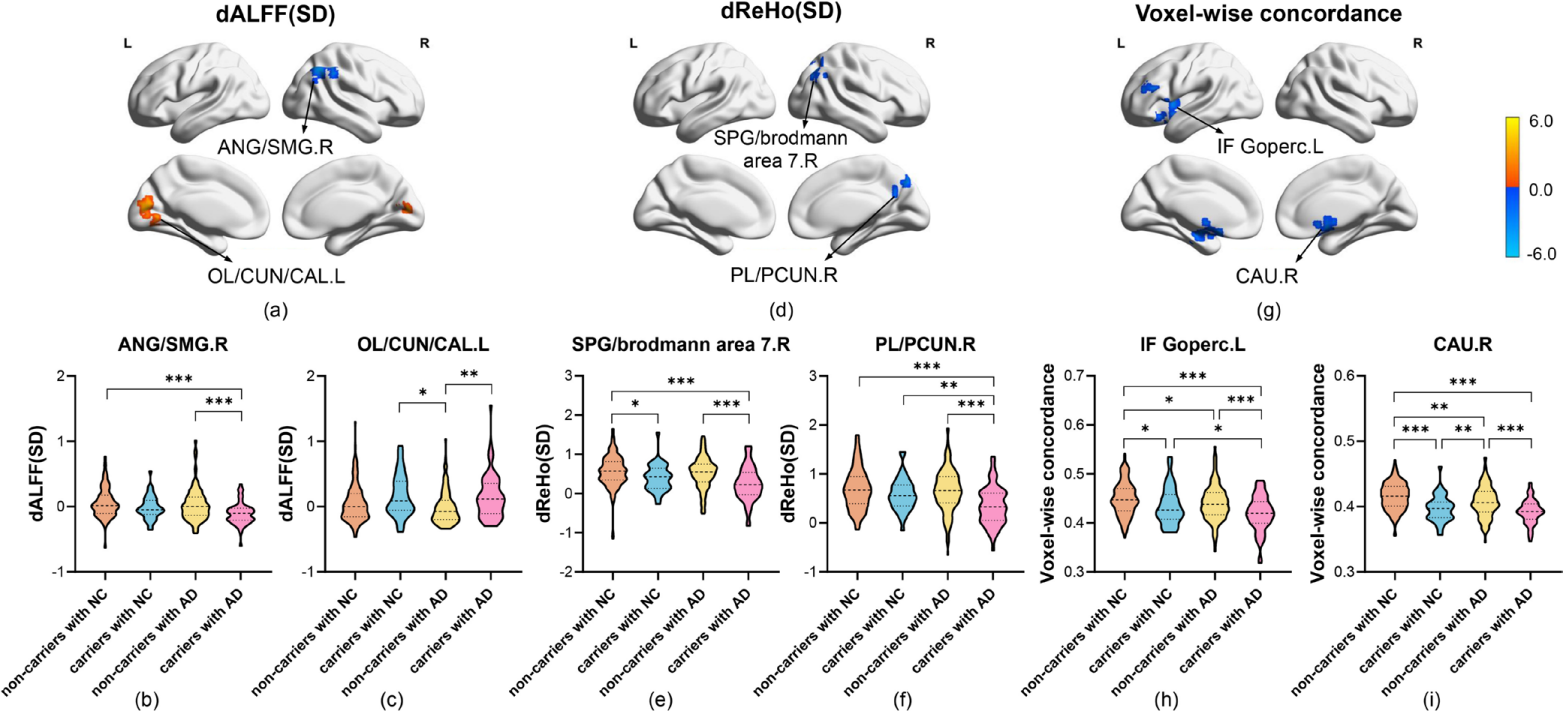
Brain regions with significant group effect differences in SD maps of dynamic metrics across the four groups. *APOE* ε4 allele led to significant differences in dALFF (SD) (a–c), dReHo (SD) (d–f), and voxel‐wise concordance (g–i) across the four groups (thresholds: For dALFF and dReHo, voxel‐level GRF < 0.001, cluster‐level GRF < 0.05, while for voxel‐wise concordance, voxel‐level GRF < 0.01, cluster‐level GRF < 0.05). The color bar indicated the T value. Warm colors indicated increased values, while cold colors indicated decreased values (Ɛ4 carriers versus non‐carriers, or AD versus NC). Age, sex, and years of education were used as covariates. *p < 0.05, **p < 0.01, ***p < 0.001.

Firstly, mixed effect analysis showed that there were significant group effects (i.e., effect of *APOE* ε4 allele) on dALFF (SD) in the right angular gyrus (ANG)/supramarginal gyrus (SMG), left occipital lobe (OL)/cuneus (CUN)/calcarine fissure and surrounding cortex (CAL) (Table 2 and Figure 1a), as well as dALFF (CV) in the right superior parietal gyrus (SPG)/precuneus (PCUN)/brodmann area 7 (Table 2 and Figure S1). There were significant group effects on dReHo (SD) in the right parietal lobe (PL)/SPG/brodmann area 7/PCUN (Table 2 and Figure 1d) and dGSCorr (Mean) in the triangular part of left inferior frontal gyrus (IF Gtriang) (Table 2 and Figure S1) as well. Secondly, brain regions with voxel‐wise concordance variability were located in the right caudate nucleus (CAU) and opercular part of left inferior frontal gyrus (IF Goperc) (Table 2 and Figure 1g). Thirdly, Table 2 exhibited cognition‐related alterations in dALFF in the right PCUN/brodmann area 7 and dfALFF in the left SPG/PCUN/brodmann area 7. Critically, *APOE* ε4 allele × cognitive status interaction emerged across groups.

**TABLE 2:** Significant differences in dynamic rs‐fMRI measures across the four groups.

Dynamic measures	Group/condition effect	Brain regions	Cluster size (voxels)	Peak MNI coordinate			Peak t value
				*x*	*y*	*z*	
dALFF (CV)	Group effect	R SPG/PCUN/brodmann area 7	84	15	−48	60	4.3350
dALFF (Mean)	Condition effect	R PCUN/brodmann area 7	71	3	−63	51	−4.5484
dALFF (SD)	Condition effect	R PCUN/brodmann area 7	117	3	−63	51	−5.2175
	Group effect	L OL/CUN/CAL	187	0	−75	12	4.7950
	Group effect	R ANG/SMG	123	51	−57	42	−4.6830
dfALFF (Mean)	Condition effect	L PCUN/brodmann area 7	55	−6	−66	54	−4.6782
dfALFF (SD)	Condition effect	L SPG/PCUN	83	−21	−60	45	−3.6881
dReHo (SD)	Group effect	R PL/PCUN	42	12	−72	42	−4.3366
	Group effect	R SPG/brodmann area 7	65	33	−63	54	−4.3213
dGSCorr (Mean)	Group effect	L IF Gtriang	154	−48	27	15	−4.3648
Voxel‐wise concordance	Group effect	R CAU	184	9	9	−9	−4.3807
	Group effect	L IF Goperc	132	−48	12	6	−4.4705

Abbreviations: ANG, angular gyrus; CAL, calcarine fissure and surrounding cortex; CAU, caudate nucleus; CUN, cuneus; dALFF, dynamic amplitude of low‐frequency fluctuations; dfALFF, dynamic fractional ALFF; dGSCorr, dynamic global signal correlation; dReHo, dynamic regional homogeneity; IF Goperc, opercular part of inferior frontal gyrus.; IF Gtriang, triangular part of inferior frontal gyrus; L, left; OL, occipital lobe; PCUN, precuneus; PL, parietal lobe; R, right; SMG, supramarginal gyrus; SPG, superior parietal gyrus.

In post hoc analysis, *APOE* ε4 carriers with AD showed decreased dALFF (SD) values in the right ANG/SMG (Figure 1b), increased dALFF (SD) values in the left OL/CUN/CAL (Figure 1c) and increased dALFF (CV) values in the right SPG/PCUN/brodmann area 7 (Figure S1) relative to non‐carriers. Similarly, *APOE* ε4 carriers with AD showed decreased dReHo (SD) values in the right PL/SPG/PCUN/brodmann area 7 in comparison with non‐carriers (Figure 1e,f). We also observed decreased dGSCorr (Mean) values in left IF Gtriang among *APOE* ε4 carriers (Figure S1). In particular, relative to non‐carriers, *APOE* ε4 carriers exhibited decreased voxel‐wise concordance in the right CAU and left IF Goperc (Figure 1h,i) independent of cognitive status.

Clusters with significant group effects and condition effects at the window step of 2 TR and the window length of 40 TR were similar to those above (Tables S1 and S2, Figures S2 and S3).

### 3.3 Effect of *APOE* ε4 Allele on Correlations Between Dynamic Rs‐fMRI Metrics and Cognition, Plasma AD Biomarkers

**FIGURE 2:**
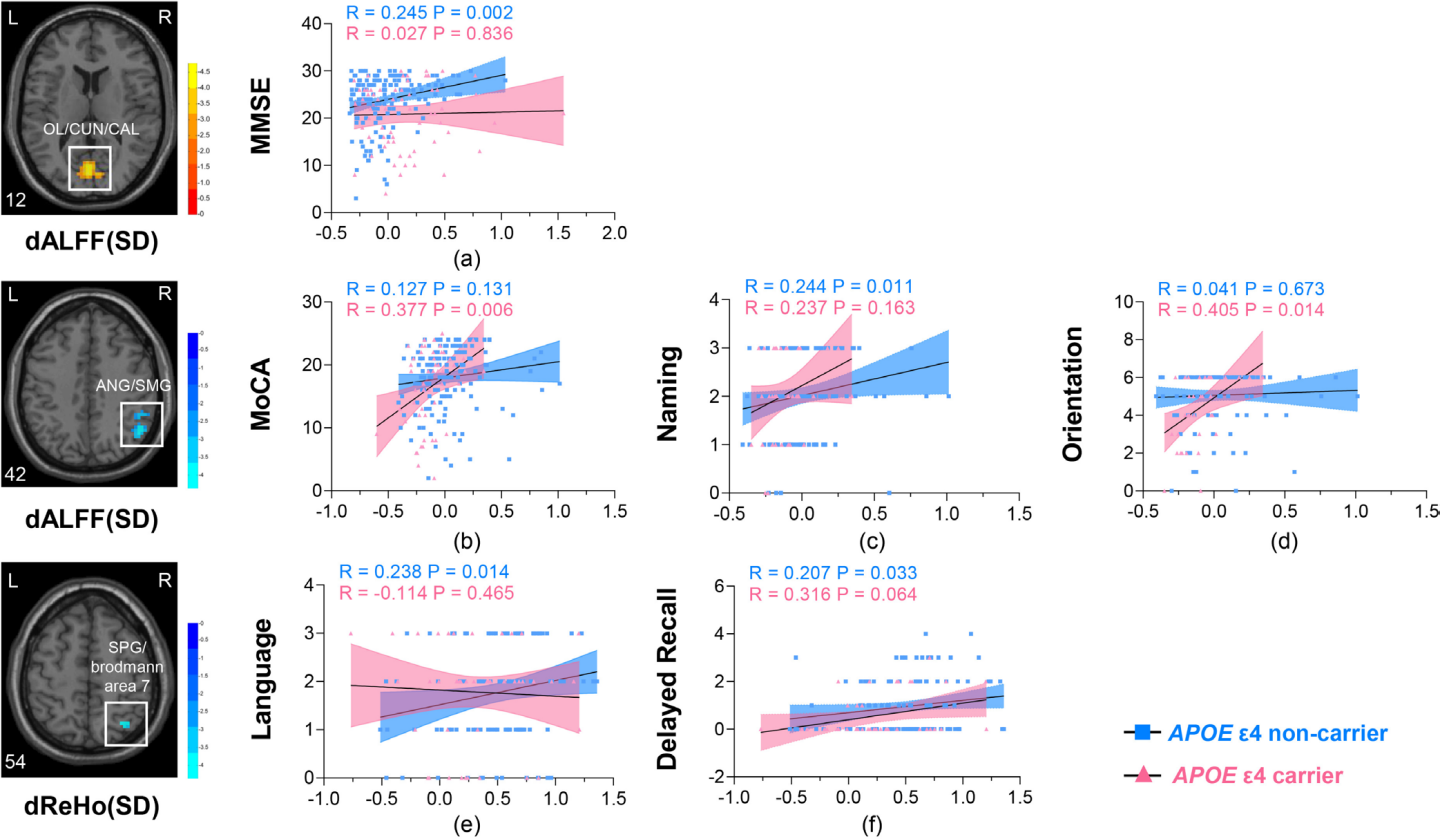
Effect of *APOE* ε4 allele on correlations between values of SD maps of dynamic rs‐fMRI metrics and cognition in the AD group. *APOE* ε4 allele weakened correlations between dALFF (SD) values in the left OL/CUN/CAL and MMSE scores (a), dALFF (SD) values in the right ANG/SMG and naming ability (c), dReHo (SD) values in the right SPG/brodmann area 7 and language function (e), delayed recall (f). While *APOE* ε4 allele enhanced correlations between dALFF (SD) values in the right ANG/SMG and MoCA scores (b), orientation function (d). Age, sex, and years of education were used as covariates. *APOE* ε4 non‐carriers and carriers were presented as blue and red colors, respectively.

According to the stratified analysis, correlations between dynamic rs‐fMRI metrics and cognition, plasma AD biomarkers among subjects with AD were associated with *APOE* ε4 mutation status while controlling for age, sex, and years of education. As shown in Figure 2, there were positive associations between dALFF (SD) values in the left OL/CUN/CAL and MMSE scores (*R* = 0.245, *p* = 0.002, Figure 2a), dALFF (SD) values in the right ANG/SMG and naming ability (*R* = 0.244, *p* = 0.011, Figure 2c), dReHo (SD) values in the right SPG/brodmann area 7 and language function (*R* = 0.238, *p* = 0.014, Figure 2e), dReHo (SD) values in the right SPG/brodmann area 7 and delayed recall (R = 0.207, *p* = 0.033, Figure 2f) in *APOE* ε4 non‐carriers rather than carriers (all *p* > 0.05). Meanwhile, dALFF (SD) values in the right ANG/SMG were positively correlated with MoCA scores (*R* = 0.377, *p* = 0.006, Figure 2b) and orientation function (*R* = 0.405, *p* = 0.014, Figure 2d) in *APOE* ε4 carriers but not in non‐carriers.

**FIGURE 3:**
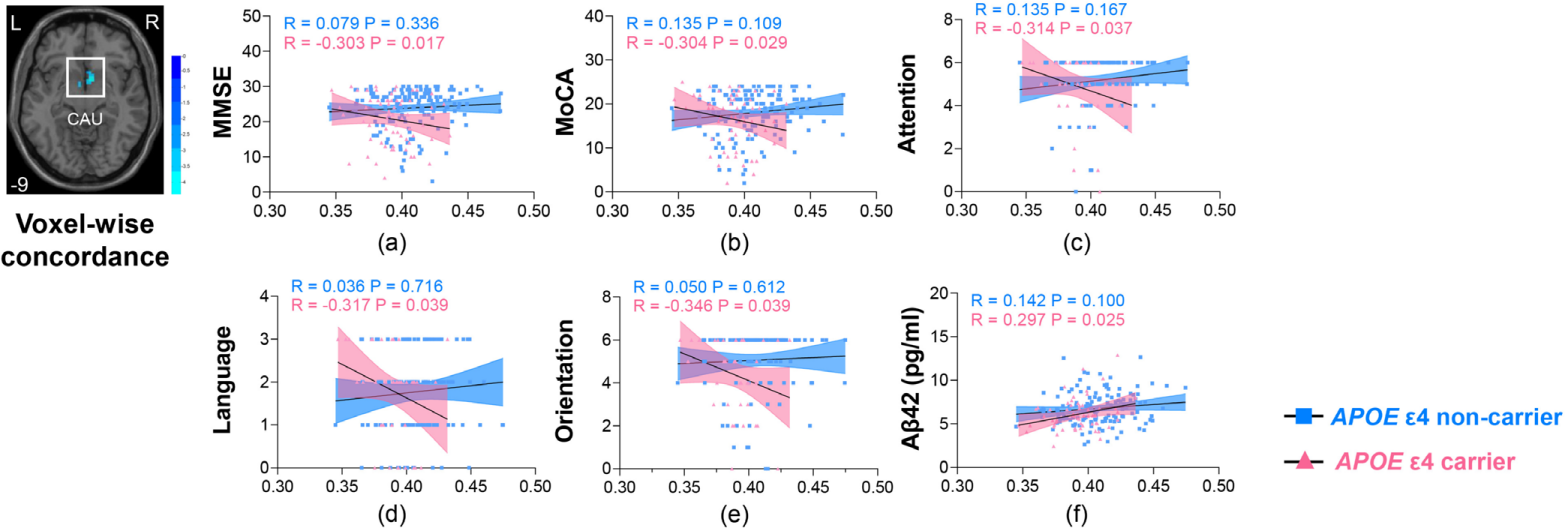
Effect of *APOE* ε4 allele on correlations between voxel‐wise concordance in the right CAU and cognition, Aβ42 in the AD group. *APOE* ε4 enhanced the correlation between voxel‐wise concordance in the right CAU and MMSE scores (a), MoCA scores (b), attention (c), language function (d), orientation function (e), plasma Aβ42 (f). Age, sex, and years of education were used as covariates. *APOE* ε4 non‐carriers and carriers were presented as blue and red colors, respectively.

Critically, we observed negative associations between voxel‐wise concordance in the right CAU and MMSE scores (*R* = −0.303, *p* = 0.017, Figure 3a), MoCA scores (*R* = −0.304, *p* = 0.029, Figure 3b), attention (*R* = −0.314, *p* = 0.037, Figure 3c), language function (*R* = −0.317, *p* = 0.039, Figure 3d), orientation function (*R* = −0.346, *p* = 0.039, Figure 3e) but a positive association with plasma Aβ42 (*R* = 0.297, *p* = 0.025, Figure 3f) in *APOE* ε4 carriers with AD rather than non‐carriers.

Most of above‐mentioned changes in correlations between dynamic metrics and cognition, Aβ42 in AD subjects within different *APOE* ε4 mutation status were also observed across multiple window parameters (Figure S4–S7).

### 3.4 Mediation Effect of Voxel‐Wise Concordance on Relationship Between Plasma Aβ42 and Language Function

**FIGURE 4:**
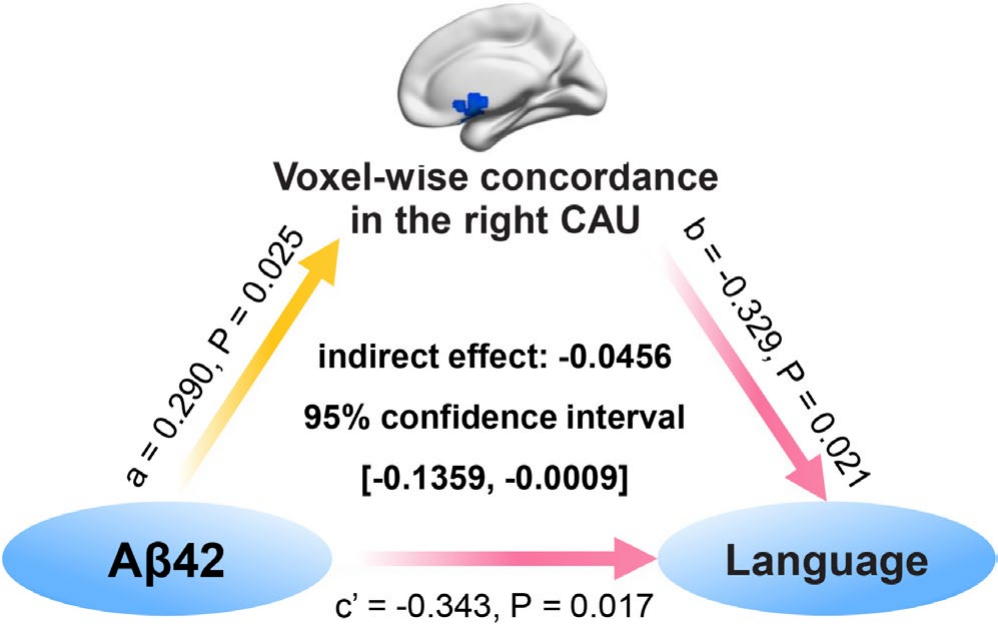
Mediation effect of voxel‐wise concordance in the right CAU on relationship between Aβ42 and language function in *APOE* ε4 carriers with AD. Voxel‐wise concordance in the right CAU partly mediated the relationship between Aβ42 and language function. The red arrow represented negative correlation, and the yellow arrow represented positive correlation. Age, sex, and years of education were used as covariates.

Bootstrapped mediation analysis exhibited that voxel‐wise concordance in the right CAU partially mediated the negative association between plasma Aβ42 and language function in *APOE* ε4 carriers with AD (indirect effect = −0.0456, 95% CI [−0.1359, −0.0009], Figure 4), after adjustment for age, sex, and years of education. This mediation pattern remained consistent across varying window parameters (Figure S8).

### 3.5 Discriminative Performance of Combined Models for AD Classification

As shown in Table 3, the k‐fold cross‐validation confirmed superior diagnostic performance of combined models in *APOE* ε4 carriers than in non‐carriers, with higher values for all performance parameters.

**TABLE 3:** The diagnostic performance of combined models in classification between NC and AD groups with a 5‐fold cross validation approach.

	Model	Cross‐validated diagnostic performance		
		Sensitivity	Specificity	Accuracy
*APOE* Ɛ4 non‐carriers	Model 1	71.61	65.63	68.57
	Model 2	80.65	65.63	73.02
	Model 3	70.97	84.38	77.78
*APOE* Ɛ4 carriers	Model 1	72.31	73.68	72.82
	Model 2	81.54	84.21	82.52
	Model 3	87.69	86.84	87.38

*Note:* Model 1: combination of age, sex, years of education, and *APOE* ε4‐related alterations in dynamic metrics; Model 2: the combination of age, sex, years of education, and *APOE* ε4‐related and cognition‐related alterations in dynamic metrics; Model 3: the combination of age, sex, years of education, AD plasma biomarkers, and *APOE* ε4‐related and cognition‐related alterations in dynamic metrics.

Further cross‐validated confusion matrices showed that among *APOE* ε4 carriers, 89.23% of AD patients were correctly identified by Model 3, 10.77% were incorrectly classified as NC individuals, and 15.79% of the NC group was misclassified. However, Model 3 showed reduced efficacy in non‐carriers, with the true positive rate of 72.26%, the false positive rate of 27.74%, the true negative rate of 80.00%, and the false negative rate of 20.00%. Model 1 and Model 2 were similar to Model 3 (Figure S9).

**FIGURE 5:**
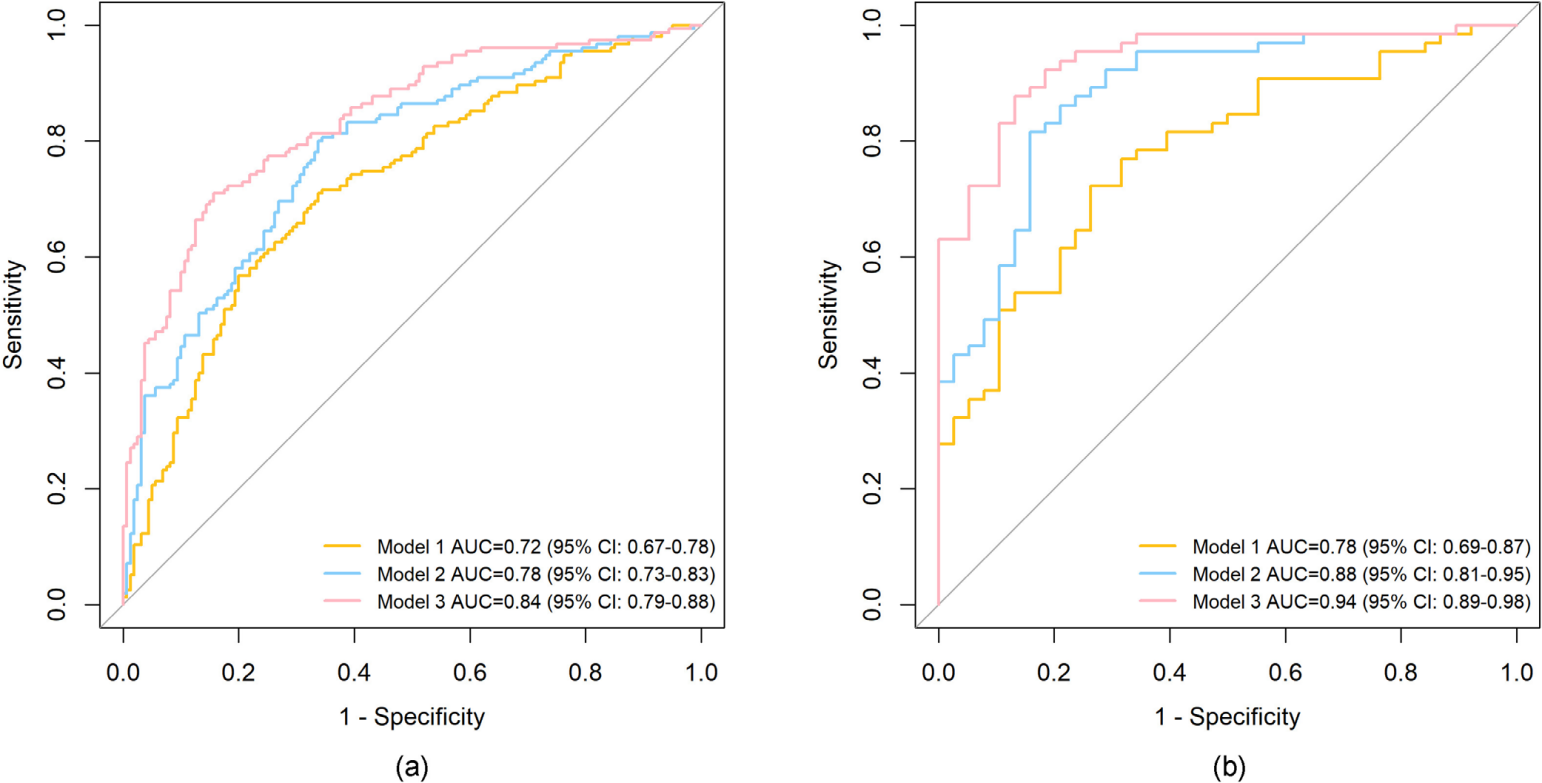
Cross‐validated ROC curves for classification between NC and AD. Combined models discriminated AD patients from NC individuals among *APOE* Ɛ4 non‐carriers (a) and *APOE* Ɛ4 carriers (b). Yellow color presented Model 1, blue color presented Model 2 and red color presented Model 3.

Also, cross‐validated ROC curves (Figure 5) showed that among *APOE* ε4 carriers, the area under the curve (AUC) of Model 1 to discriminate AD patients from NC individuals was 0.78 (95% CI [0.69, 0.87]), the AUC of Model 2 was 0.88 (95% CI [0.81, 0.95]), and the AUC of Model 3 was up to 0.94 (95% CI [0.89, 0.98]). Performance was consistently lower in non‐carriers.

## 4 Discussion

To the best of our knowledge, this is the first study to comprehensively examine alterations in dynamic local spontaneous brain activity and functional integration associated with *APOE* ε4 allele in AD. We found that *APOE* ε4 allele impacted dALFF, dReHo, and dGSCorr including frontal, parietal, and occipital lobes, as well as voxel‐wise concordance in the right CAU and left IF Goperc. The *APOE* ε4 allele influenced correlations of dynamic functional activities with both cognition and Aβ pathology in AD. Furthermore, the voxel‐wise concordance in the right CAU partially mediated the relationship between Aβ42 and language function in *APOE* ε4 carriers with AD. Finally, the dynamic combined model could effectively identified cognitive status, particularly in *APOE* ε4 carriers. This in‐depth analysis of dynamic local activity, concordance, and plasma AD pathology may provide novel insights into the mechanisms of *APOE* ε4 in AD pathology.

The present study revealed that *APOE* ε4 carriers with AD exhibited spatial overlap of dALFF and dReHo alterations in the right SPG/PCUN/brodmann area 7, suggesting increased variability in neural activity intensity and decreased stability of synchronous activity in adjacent voxels in these regions. Previous studies have observed abnormal ALFF and ReHo in these regions in amnestic MCI (Pan et al. 2017; Zhen 2018; Zhong et al. 2022) and subjective cognitive decline (Li et al. 2021), further indicating that aberrant spontaneous brain activity in the right SPG/PCUN/brodmann area 7 may be associated with cognitive decline. Our further analysis showed that dReHo in the right SPG/brodmann area 7 positively correlated with language function and delayed recall in *APOE* ε4 non‐carriers but not in carriers. Given that SPG is associated with voice severity in essential tremor (De Lima Xavier and Simonyan 2020), auditory integration for vocal pitch regulation (Chen et al. 2021), and long‐term delayed recall (Ma et al. 2023), we speculate that *APOE* ε4 allele may cause inflexible connectivity between the right SPG/PCUN/brodmann area 7 and neighboring brain regions during the integration, processing, and analysis of cognitive information in AD patients.

Nevertheless, the anatomical distributions of changes in dynamic local metrics were not completely overlapping, indicating that *APOE* ε4 allele may affect brain activity by physiological complementarity in different regions (Chen et al. 2023). First, the decreased dALFF (SD) of the right ANG/SMG in *APOE* ε4 carriers suggested that *APOE* ε4 allele weakens the stability of neuronal activity in the inferior parietal lobule (IPL). The IPL plays a key role in object recognition, social cognition, language function, and orientation (Binder and Desai 2011; Peer et al. 2015). Wei et al. also reported decreased mean dALFF in the IPL in subjective cognitive decline (Wei et al. 2022). Since higher dALFF stability contributes to flexible brain adaptability to environmental demands (Liao et al. 2019), our partial correlation analysis indicated that worse general cognition and orientation function in *APOE* ε4 carriers with AD likely result from inflexible neuronal activity in the right IPL during time‐varying intrinsic processing. Second, dALFF stability in the left OL/CUN/CAL increased in *APOE* ε4 carriers. Given the critical role of the OL in various higher cognitive functions (Bettencourt and Xu 2016; Snytte et al. 2022), we speculated that *APOE* ε4 allele may cause a compensatory increase in neural flexibility that disrupts the relationship between neural stability and general cognition in this region. These regions represent potential therapeutic targets for AD patients carrying *APOE* ε4 allele.

The voxel‐wise concordance was then used to quantify the harmony of dynamic local metrics during state transitions. Results revealed decreased voxel‐wise concordance in the left IF Goperc and right CAU in *APOE* ε4 carriers, potentially due to inconsistent synchronization among dynamic local metrics. The IF Goperc constitutes part of Broca's area and serves as the language network hub connecting diverse cortical, subcortical, and cerebellar regions (Bulut 2022). A rs‐fMRI study based on visibility graph demonstrated that left IF Goperc plays a significant role in ad (Gao et al. 2020 ). However, our results indicated that decreased concordance in the left IF Goperc associated with *APOE* ε4 did not significantly affect cognitive function in AD patients.

Our correlation analysis suggested that the *APOE* ε4 allele strengthened the correlation between functional integration in the right CAU and cognition. As a key component of the cortico‐basal ganglia‐thalamo‐cortical circuit (Kim, Kim, et al. 2022), the CAU affects multiple cognitive functions including attention and language function (Herman et al. 2020; Tomasi and Volkow 2012). Prior studies have observed functional and structural caudate alterations in *APOE* ε4 carriers. For example, reduced hippocampal‐caudate FC in *APOE* ε4 carriers correlates significantly with episodic memory impairment (Li et al. 2014). Additionally, the *APOE* ε4 allele accelerates gray matter atrophy in the CAU among patients with MCI to AD conversion (Spampinato et al. 2011). Unexpectedly, we observed that higher voxel‐wise concordance was associated with worse cognition in *APOE* ε4 carriers with AD, which may be explained by synchronous decreases in all dynamic local metrics as AD severity progresses.

Furthermore, we found that voxel‐wise activity in the right CAU was positively associated with plasma Aβ42 exclusively in *APOE* ε4 carriers. Previous studies have indicated that *APOE* ε4 carriers exhibited accelerated Aβ deposition and significant degeneration of serotonergic synapses in the ventromedial caudate nucleus (Kim, Chun, et al. 2022; Postupna et al. 2017; Ichimata et al. 2024). Notably, Aβ positivity in the caudate nucleus was significantly correlated with global cognitive decline in *APOE* ε4 carriers (Brugulat‐Serrat et al. 2023), aligning with our findings. Our mediation analysis further indicated that plasma Aβ42 acts as an upstream driver of functional alterations in the right CAU. Researchers have reported an interactive effect between smoking and functional connectivity of the right CAU with the bilateral anterior cingulate cortex on language function in MCI patients due to ad (Qiu et al. 2022 ). Furthermore, the bilateral CAU was proven to be associated with advanced semantic fluency strategies, particularly during the MCI stage (Ahn et al. 2022; Kwak et al. 2022). Integrating our mediation model results with these prior findings, we propose that the right CAU critically influences language function in AD. However, spatial relationships between voxel‐wise concordance and Aβ deposition require validation via positron emission tomography, and causal relationships among these factors need longitudinal investigation.

Ultimately, ROC analysis demonstrated that these dynamic metrics could serve as potential early imaging biomarkers and prognostic markers for AD, and the corresponding brain regions may represent effective targets for neuroregulatory therapy in early AD.

Several limitations should be mentioned in the present study. First, recent studies suggest that *APOE* ε4 homozygotes may have distinct functional and pathological trajectories, requiring separate analysis from heterozygotes (Fortea 2024). Due to the substantial sample size disparity between these groups, we did not conduct subgroup analysis, limiting this study's applicability for screening or diagnostic purposes. We will improve this shortcoming in the following research. Second, given that dfALFF, dReHo, and dGSCorr are more developed in characterizing local intrinsic activity, only these three dynamic local metrics were applied to calculate voxel‐wise concordance. Nevertheless, the optimal combination for comprehensive characterization remains undetermined. Third, as a cross‐sectional study with a limited sample size, we are continuing to recruit new subjects and follow up with them to validate our findings.

## 5 Conclusion

In conclusion, our findings indicated that the *APOE* ε4 allele broadly influenced dynamic local spontaneous brain activity and functional integration in AD. It also modified the relationships between alterations in dynamic metrics and both cognition and plasma AD biomarkers. Importantly, voxel‐wise concordance in the right CAU partially mediated the relationship between plasma Aβ and language function. Our research highlights the effects of *APOE* ε4 on dynamic metric‐cognition relationships in AD, providing new insights into pathological mechanisms. Moreover, functionally altered regions may suggest therapeutic targets for AD patients with *APOE* ε4 allele. However, the cross‐sectional design and lack of subgroup analysis limit the application potential of this study in screening or diagnosis.

## Author Contributions

**Yi Tan:** conceptualization, methodology, formal analysis, data curation, and writing – original draft; **Dan Yang:** conceptualization, methodology, and writing – review and editing; **Zhihong Ke:** data curation; **Zheqi Hu:** data curation; **Wenting Song:** data curation and formal analysis; **Limoran Tang:** data curation; **Zhixin Zhou:** data curation; **Yuting Mo:** data curation; **Lili Huang:** data curation; **Yun Xu:** supervision, project administration, and funding acquisition.

## Conflicts of Interest

The authors declare no conflicts of interest.

## Supporting information


Data S1.

## Data Availability

The data that support the findings of this study are available from the corresponding author upon reasonable request. The data are not publicly available due to privacy or ethical restrictions.
