# Supplementary material for: The Effect of APOE ε4 Allele on Dynamic Local Spontaneous Brain Activity and Functional Integration in Alzheimer's Disease
Source: Hum Brain Mapp. 2025 Jul 11;46(10):e70269. doi: 10.1002/hbm.70269 (PMC12246834; doi:10.1002/hbm.70269)
Supplement: Supplementary file 1 — Data S1. [file HBM-46-e70269-s001.docx]

**SUPPLEMENTAL TABLES**

**Supplemental Table 1. Significant differences in dynamic rs-fMRI metrics across the four groups** **(window length 30TR, window step 2TR).**

| **Dynamic metrics** | **Group/Condition effect** | **Brain regions** | **Cluster size**  **(voxels)** | **Peak MNI coordinate** | | | **Peak T value** |
| --- | --- | --- | --- | --- | --- | --- | --- |
|  |  |  |  | **x** | **y** | **z** |  |
| dALFF (CV) | Group effect | R SPG/PCUN/brodmann area 7 | 85 | 15 | -48 | 60 | 4.337 |
| dALFF (Mean) | Condition effect | R PCUN/brodmann area 7 | 71 | 3 | -63 | 51 | -4.551 |
| dALFF (SD) | Condition effect | R PCUN/brodmann area 7 | 117 | 3 | -63 | 51 | -5.219 |
|  | Group effect | L OL/CUN/CAL | 179 | 0 | -75 | 12 | 4.791 |
|  | Group effect | R ANG/SMG | 121 | 51 | -57 | 42 | -4.685 |
| dfALFF (Mean) | Condition effect | L PCUN/brodmann area 7 | 55 | -6 | -66 | 54 | -4.673 |
| dfALFF (SD) | Condition effect | L SPG/PCUN | 74 | -21 | -60 | 45 | -3.700 |
| dReHo (SD) | Group effect | R PL/PCUN | 46 | 12 | -72 | 42 | -4.342 |
|  | Group effect | R SPG/brodmann area 7 | 65 | 33 | -63 | 54 | -4.321 |
| dGSCorr (Mean) | Group effect | L IF Gtriang | 156 | -48 | 27 | 15 | -4.363 |
| Voxel-wise concordance | Group effect | R CAU | 183 | 9 | 9 | -9 | -4.376 |
|  | Group effect | L IF Goperc | 131 | -48 | 12 | 6 | -4.464 |

Abbreviations: dALFF, dynamic amplitude of low-frequency fluctuations; dfALFF, dynamic fractional ALFF; dReHo, dynamic regional homogeneity; dGSCorr, dynamic global signal correlation; L, left; R, right; SPG, superior parietal gyrus; PCUN, precuneus; OL, occipital lobe; CUN, cuneus; CAL, calcarine fissure and surrounding cortex; ANG, angular gyrus; SMG, supramarginal gyrus; PL, parietal lobe; IF Gtriang, triangular part of inferior frontal gyrus; CAU, caudate nucleus; IF Goperc, opercular part of inferior frontal gyrus.

**Supplemental Table 2. Significant differences in dynamic rs-fMRI metrics across the four groups** **(window length 40TR, window step 1TR).**

| **Dynamic metrics** | **Group/Condition effect** | **Brain regions** | **Cluster size**  **(voxels)** | **Peak MNI coordinate** | | | **Peak T value** |
| --- | --- | --- | --- | --- | --- | --- | --- |
|  |  |  |  | **x** | **y** | **z** |  |
| dALFF (CV) | Group effect | R MFG/SFGdor | 71 | 24 | -6 | 54 | -4.070 |
|  | Group effect | R SPG/PCUN/brodmann area 7 | 61 | 15 | -48 | 60 | 4.189 |
| dALFF (Mean) | Condition effect | R PCUN/brodmann area 7 | 70 | 3 | -63 | 51 | -4.549 |
| dALFF (SD) | Condition effect | R PCUN | 112 | 3 | -66 | 51 | -5.238 |
|  | Group effect | L OL/CUN/CAL | 203 | 0 | -75 | 12 | 4.810 |
| dfALFF (CV) | Group effect | L ITG/brodmann area 19 | 72 | -48 | -60 | -6 | -4.152 |
| dfALFF (Mean) | Condition effect | R PL | 44 | 33 | -63 | 30 | -4.707 |
|  | Condition effect | R PCUN/brodmann area 7 | 55 | 3 | -63 | 51 | -4.665 |
| dfALFF (SD) | Condition effect | L PCUN | 68 | -9 | -66 | 42 | -3.876 |
| dReHo (SD) | Group effect | R PCUN | 66 | 15 | -72 | 42 | -4.453 |
|  | Group effect | R PCUN/ANG | 95 | 39 | -72 | 36 | -4.354 |
| dGSCorr (Mean) | Group effect | L PHG | 111 | -27 | -6 | -27 | -4.139 |
|  | Group effect | L IF Gtriang | 143 | -48 | 27 | 15 | -4.344 |
| dGSCorr (SD) | Group effect | R CUN/SOG | 102 | 24 | -90 | 24 | -4.748 |
| Voxel-wise concordance | Condition effect | R FL/PoCG/brodmann area 6 | 151 | 66 | -6 | 30 | 4.430 |
|  | Group effect | R CAU | 160 | 9 | 9 | -9 | -4.146 |

Abbreviations: dALFF, dynamic amplitude of low-frequency fluctuations; dfALFF, dynamic fractional ALFF; dReHo, dynamic regional homogeneity; dGSCorr, dynamic global signal correlation; L, left; R, right; MFG, middle frontal gyrus; SFGdor, dorsolateral of superior frontal gyrus; SPG, superior parietal gyrus; PCUN, precuneus; OL, occipital lobe; CUN, cuneus; CAL, calcarine fissure and surrounding cortex; ITG, inferior temporal gyrus; ANG, angular gyrus; PL, parietal lobe; PHG, parahippocampal gyrus; IF Gtriang, triangular part of inferior frontal gyrus; SOG, superior occipital gyrus; FL, frontal lobe; PoCG, postcentral gyrus; CAU, caudate nucleus.

**SUPPLEMENTAL FUGIRES**

**
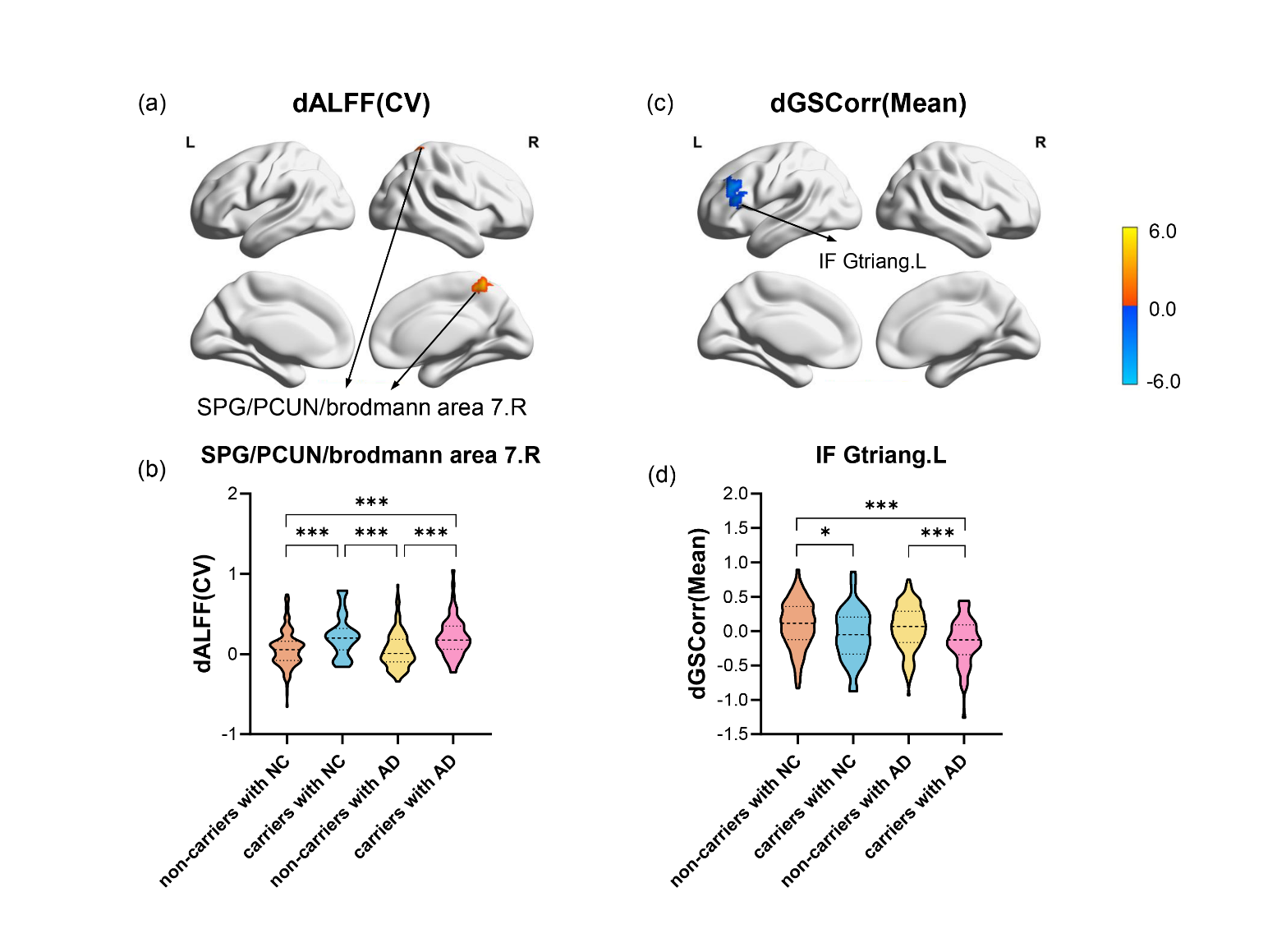
**

**Supplemental Figure 1. Brain regions with significant group effect differences in CV map of dALFF and Mean map of dGSorr across the four groups.** *APOE* ε4 allele led to significant differences in dALFF (CV) **(a-b)** and dGSCorr (Mean) **(c-d)** across the four groups (thresholds: voxel-level GRF < 0.001, cluster-level GRF < 0.05). The color bar indicated the T value. Warm colors indicated increased values, while cold colors indicated decreased values (Ɛ4 carriers versus non-carriers, or AD versus NC). Age, sex, and years of education were used as covariates. * P < 0.05, *** P < 0.001.


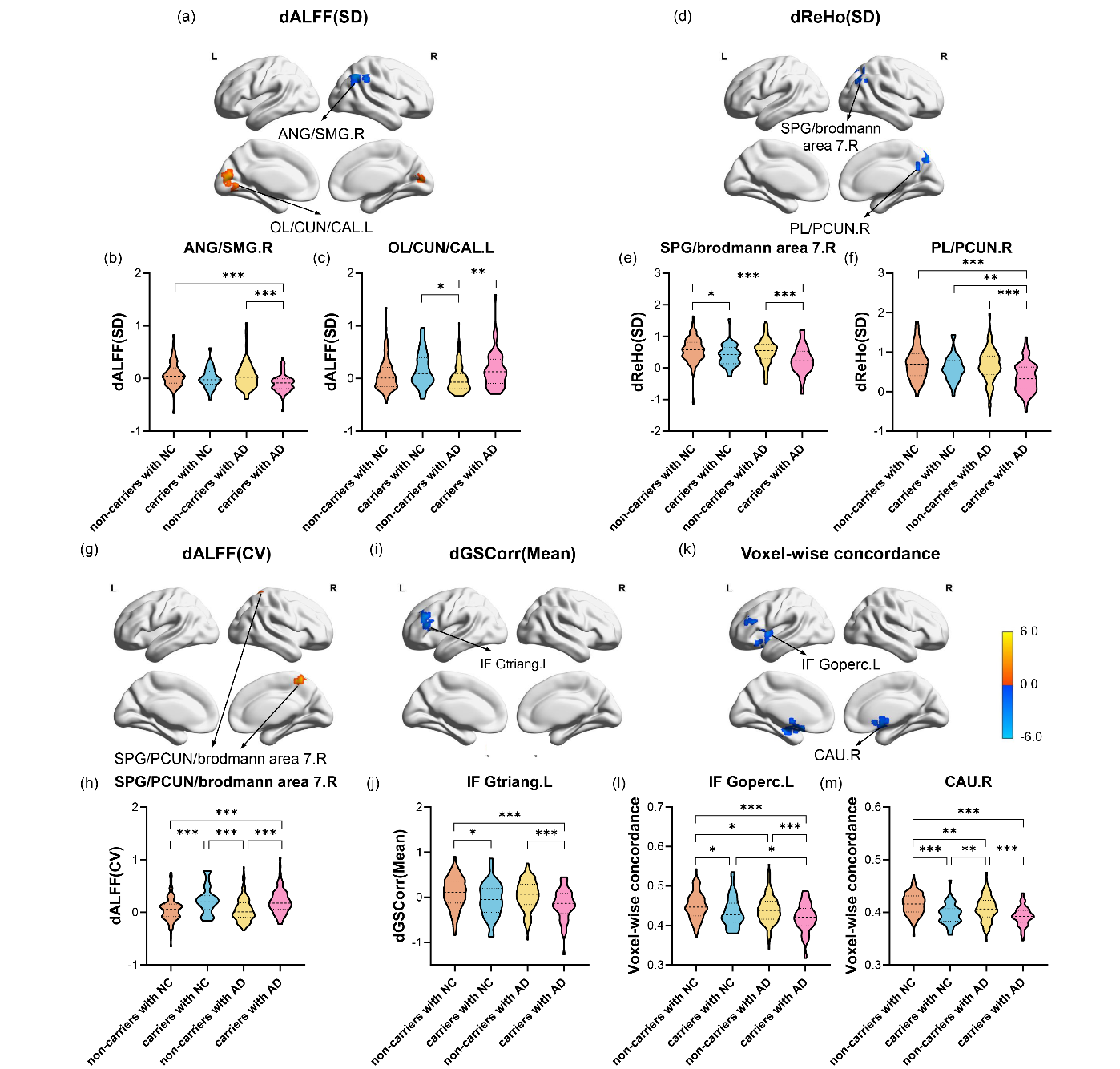


**Supplemental Figure 2. Brain regions with significant group effect differences in dynamic metrics across the four groups (window length 30TR, window step 2TR).** *APOE* ε4 allele led to significant differences in dynamic local metrics and voxel-wise concordance across the four groups (thresholds: voxel-level GRF < 0.001 or 0.01, cluster-level GRF < 0.05). The color bar indicated the T value. Warm colors indicated increased values, while cold colors indicated decreased values (Ɛ4 carriers versus non-carriers, or AD versus NC). Age, sex, and years of education were used as covariates. * P < 0.05, ** P < 0.01, *** P < 0.001.


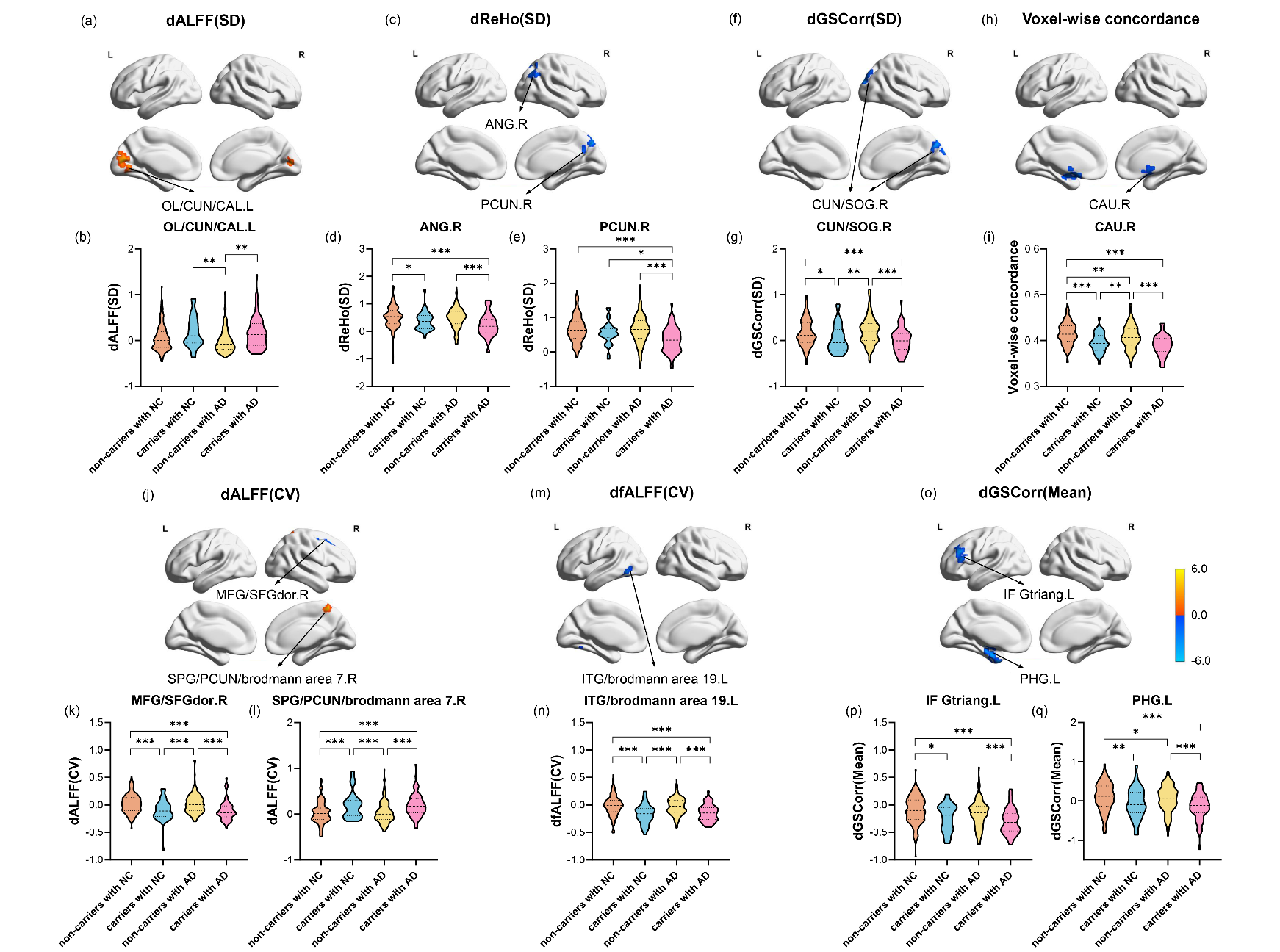


**Supplemental Figure 3. Brain regions with significant group effect differences in dynamic metrics across the four groups (window length 40TR, window step 1TR).** *APOE* ε4 allele led to significant differences in dynamic local metrics and voxel-wise concordance across the four groups (thresholds: voxel-level GRF < 0.001 or 0.01, cluster-level GRF < 0.05). The color bar indicated the T value. Warm colors indicated increased values, while cold colors indicated decreased values (Ɛ4 carriers versus non-carriers, or AD versus NC). Age, sex, and years of education were used as covariates. * P < 0.05, ** P < 0.01, *** P < 0.001.


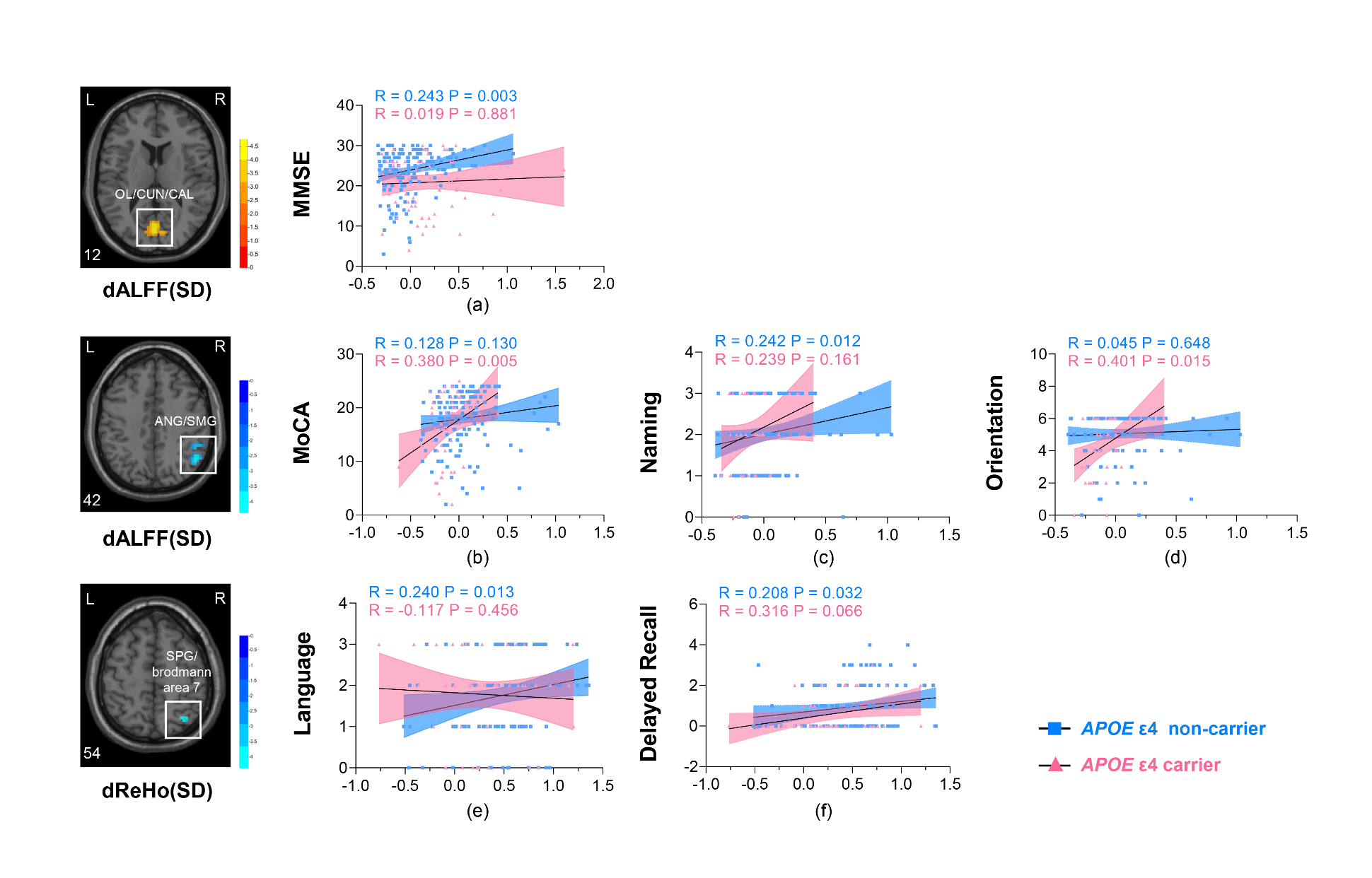


**Supplemental Figure 4. Effect of *APOE* ε4 allele on correlations between values of SD maps of dynamic rs-fMRI metrics and cognition in the AD group (window length 30TR, window step 2TR). (a-d)** Correlations between dALFF (SD) values and general cognition, naming ability, orientation function. **(e-f)** Correlations between dReHo (SD) values and language function, delayed recall. Age, sex, and years of education were used as covariates. *APOE* ε4 non-carriers and carriers were presented as blue and red colors, respectively.


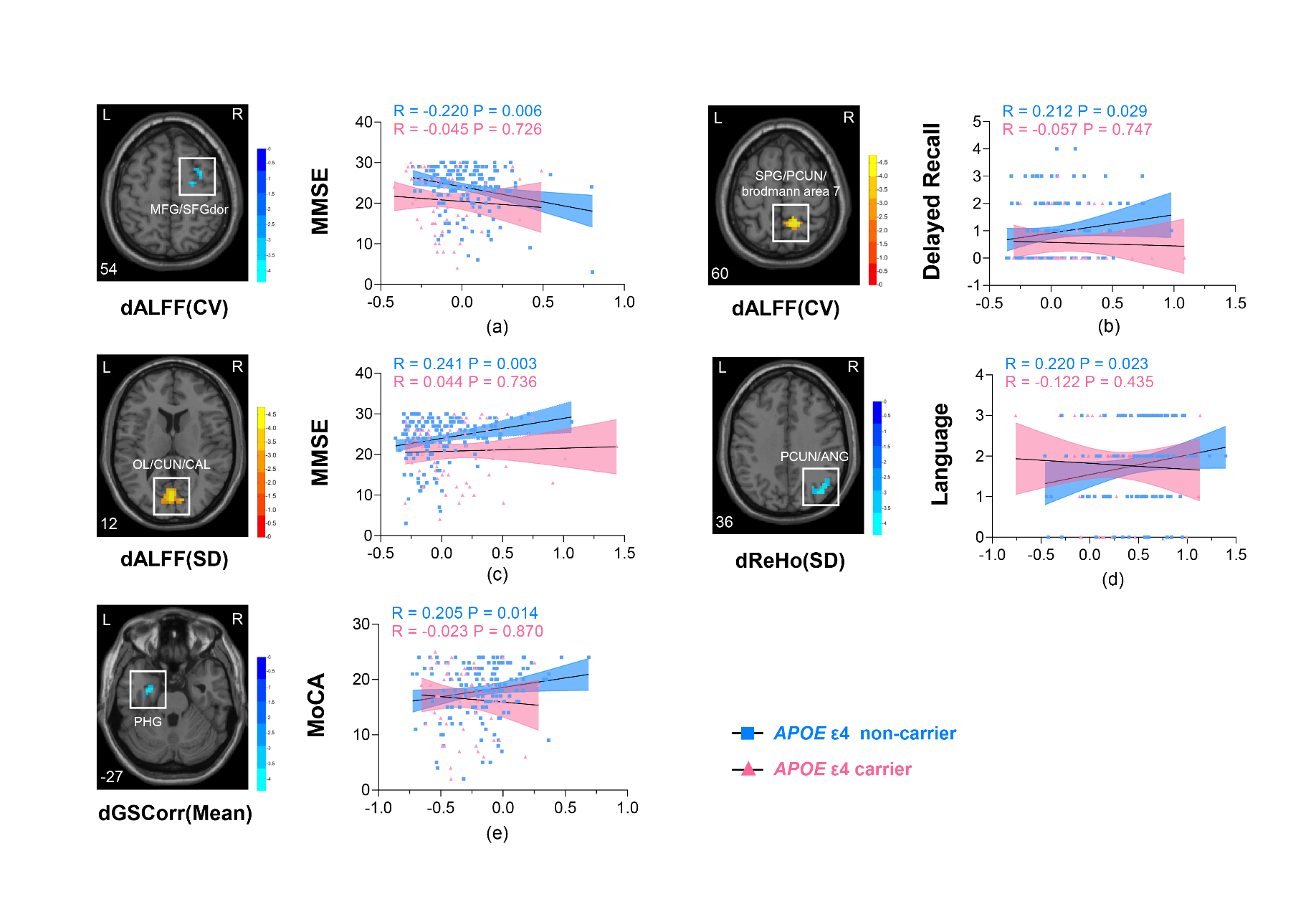


**Supplemental Figure 5. Effect of *APOE* ε4 allele on correlations between values of dynamic rs-fMRI metrics and cognition in the AD group (window length 40TR, window step 1TR). (a-c)** Correlations between dALFF values and MMSE scores, delayed recall. **(d)** Correlation between dReHo values and language function. **(e)** Correlation between dGSCorr values and MoCA scores. Age, sex, and years of education were used as covariates. *APOE* ε4 non-carriers and carriers were presented as blue and red colors, respectively.


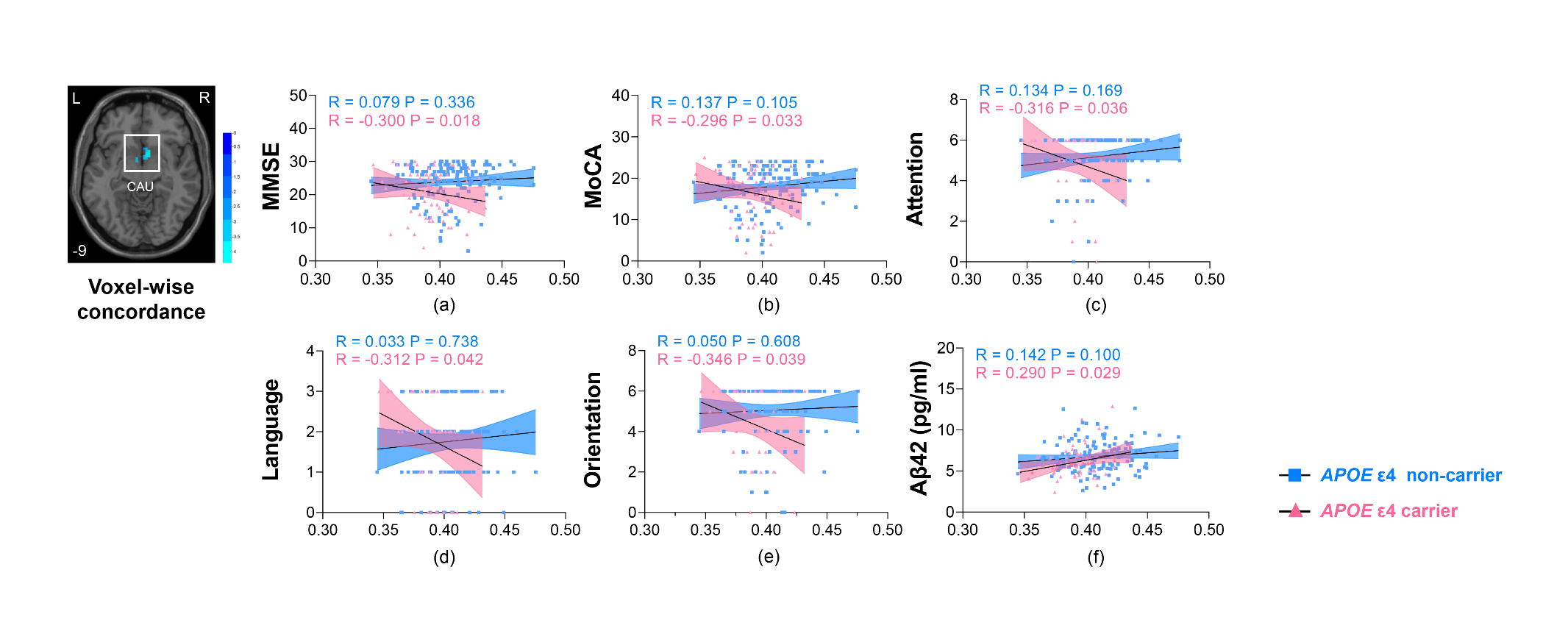


**Supplemental Figure 6. Effect of *APOE* ε4 allele on correlations between voxel-wise concordance in the right CAU and cognition, Aβ42 in the AD group (window length 30TR, window step 2TR).** *APOE* ε4 allele enhanced correlations between voxel-wise concordance in the right CAU and MMSE scores **(a)**, MoCA scores **(b)**, attention **(c)**, language function **(d)**, orientation function **(e)**, plasma Aβ42 **(f)**. Age, sex, and years of education were used as covariates. *APOE* ε4 non-carriers and carriers were presented as blue and red colors, respectively.


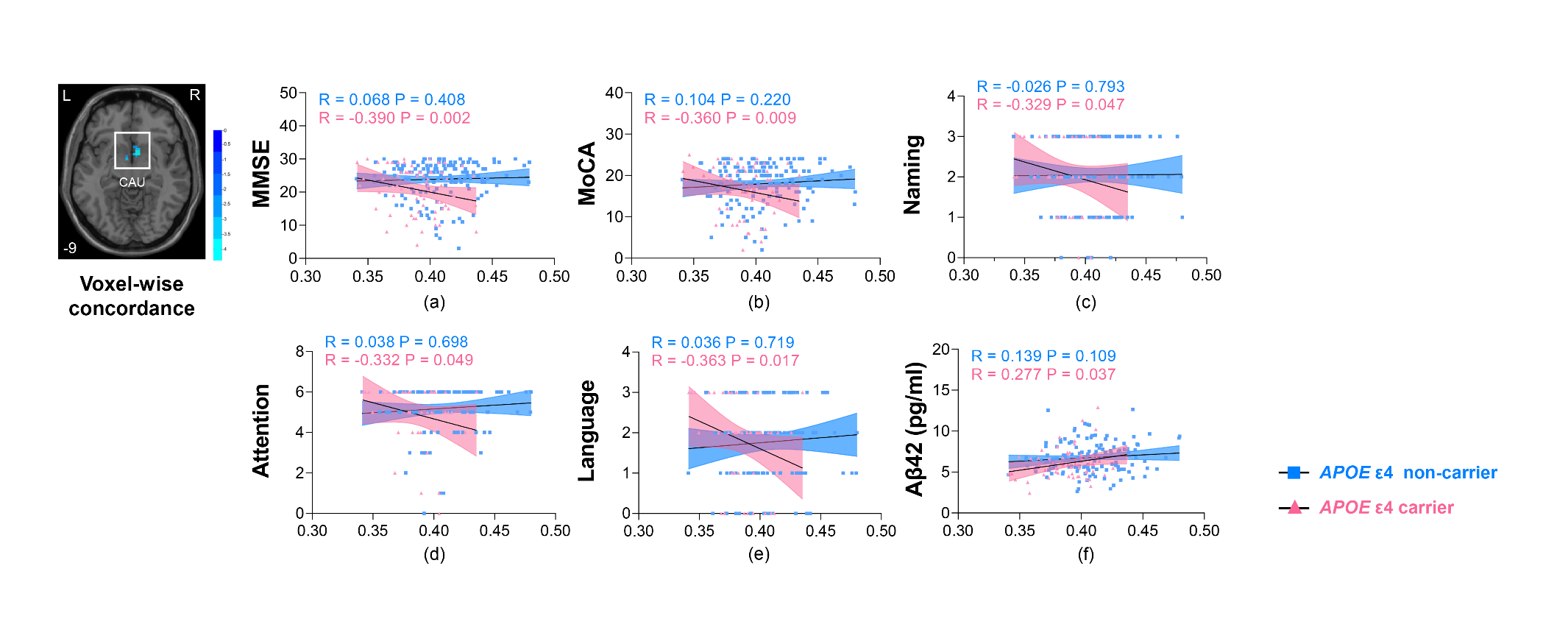


**Supplemental Figure 7. Effect of *APOE* ε4 allele on correlations between voxel-wise concordance in the right CAU and cognition, Aβ42 in the AD group (window length 40TR, window step 1TR).** *APOE* ε4 allele enhanced correlations between voxel-wise concordance in the right CAU and MMSE scores **(a)**, MoCA scores **(b)**, naming ability **(c)**, attention **(d)**, language function **(e)**, plasma Aβ42 **(f)**. Age, sex, and years of education were used as covariates. *APOE* ε4 non-carriers and carriers were presented as blue and red colors, respectively.


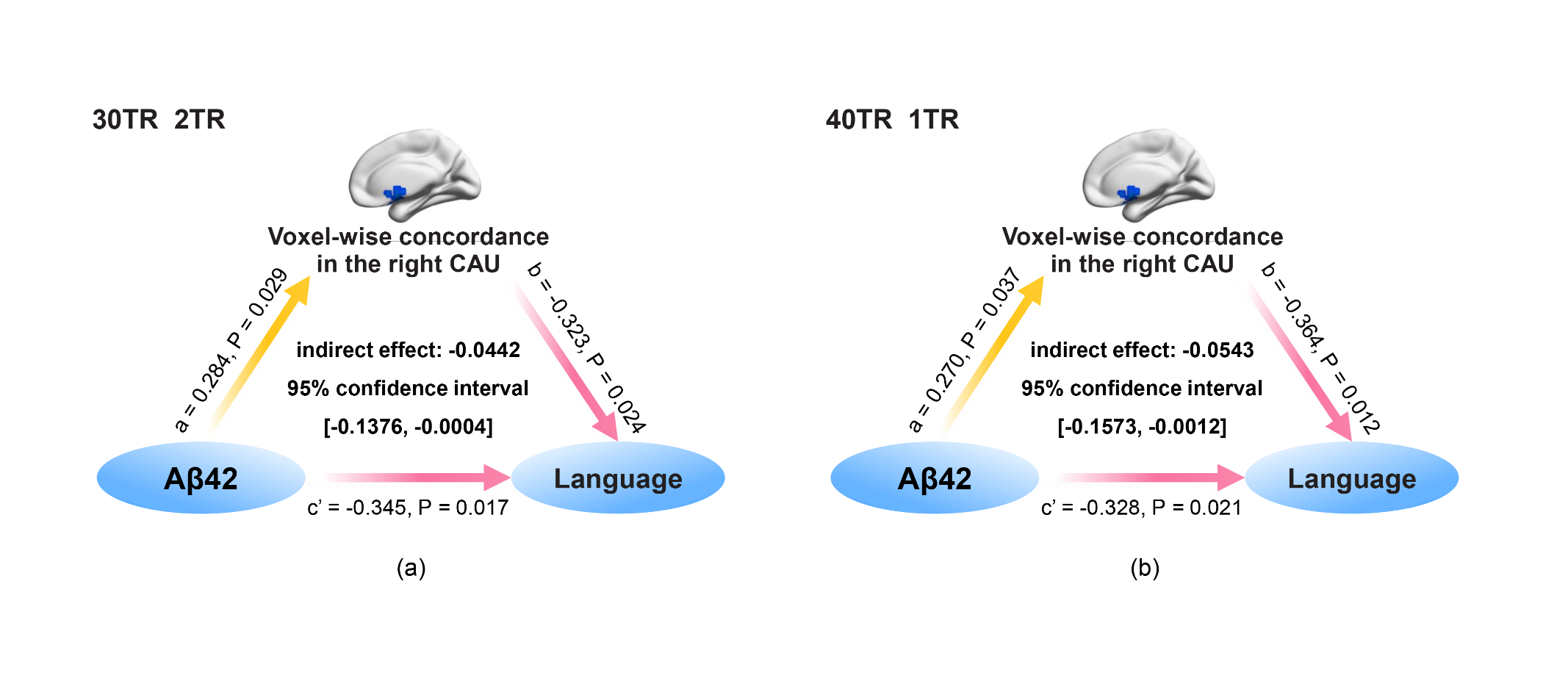


**Supplemental Figure 8. Mediation effect of voxel-wise concordance in the right CAU on relationship between Aβ42 and language function in *APOE* ε4 carriers with AD (window length 30TR, window step 2TR and window length 40TR, window step 1TR).** Voxel-wise concordance in the right CAU mediated the relationship between Aβ42 and language function at window length 30TR, window step 2TR **(a)** and window length 40TR, window step 1TR **(b)**. The pink arrows represented negative correlation, and the yellow arrows represented positive correlation. Age, sex, and years of education were used as covariates.

**
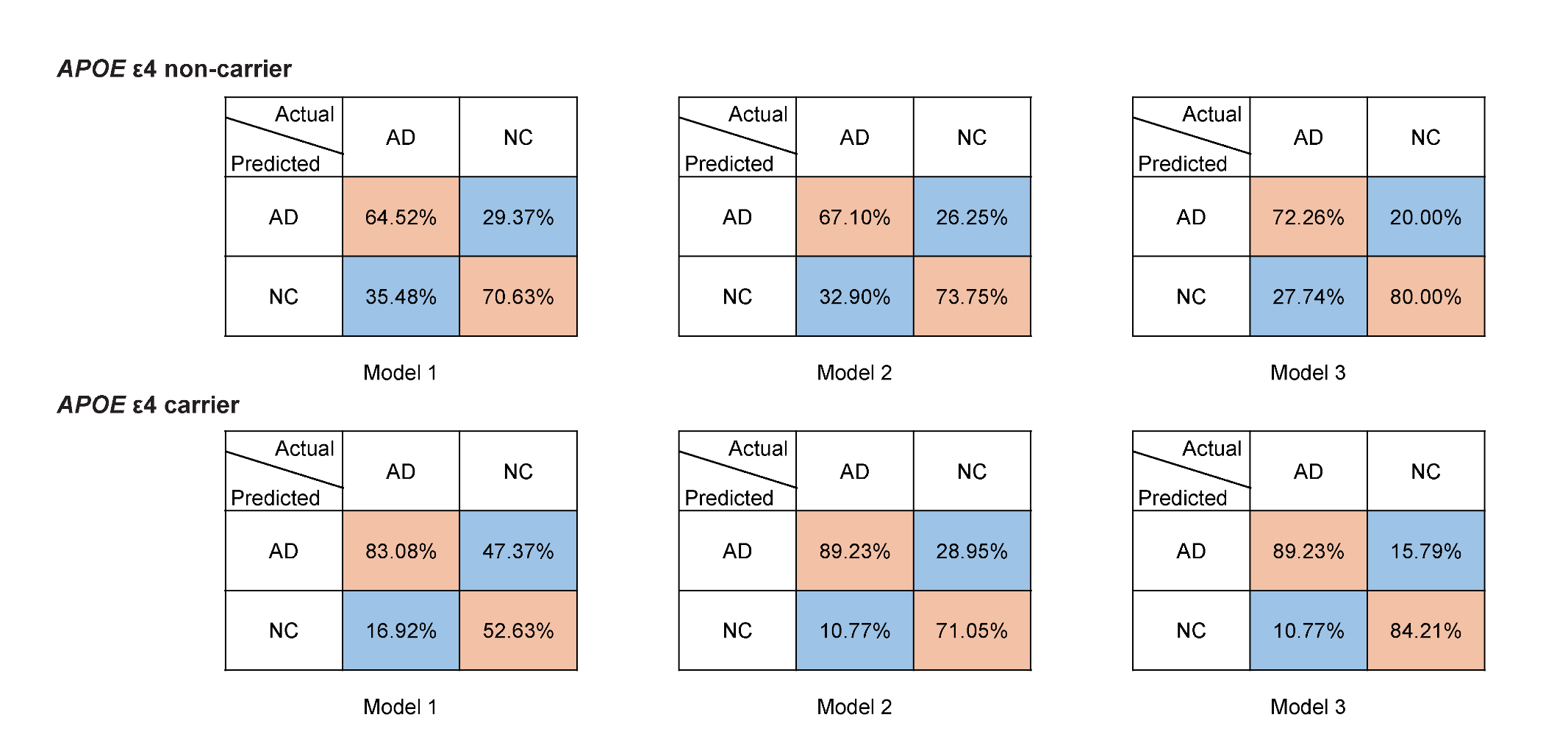
**

**Supplemental Figure 9. Cross-validated confusion matrices for classification between NC and AD.** Combined models discriminated AD patients from NC individuals among *APOE* Ɛ4 non-carriers and carriers. Model 1: combination of age, sex, years of education, and *APOE* ε4-related alterations in dynamic metrics; Model 2: the combination of age, sex, years of education, and *APOE* ε4-related and cognition-related alterations in dynamic metrics; Model 3: the combination of age, sex, years of education, AD plasma biomarkers, and *APOE* ε4-related and cognition-related alterations in dynamic metrics.
